# Targeted radionuclide therapy in endocrine-related cancers: advances in the last decade

**DOI:** 10.3389/fendo.2023.1187870

**Published:** 2023-11-20

**Authors:** Taymeyah Al-Toubah, Jonathan Strosberg, Julie Hallanger-Johnson, Ghassan El-Haddad

**Affiliations:** ^1^ Department of GI Oncology, H. Lee Moffitt Cancer Center & Research Institute, Tampa, FL, United States; ^2^ Department of Head and Neck - Endocrine Oncology, H. Lee Moffitt Cancer Center & Research Institute, Tampa, FL, United States; ^3^ Department of Radiology, H. Lee Moffitt Cancer Center & Research Institute, Tampa, FL, United States; ^4^ Department of Nuclear Medicine, H. Lee Moffitt Cancer Center & Research Institute, Tampa, FL, United States

**Keywords:** neuroendocrine, endocrine, pheochromocytoma, paraganglioma, radiopharmaceutical

## Abstract

Targeted radionuclide therapy plays an increasingly important role in managing endocrine-related tumors and significantly advances the therapeutic landscape for patients with these diseases. With increasing FDA-approved therapies and advances in the field, come an increased knowledge of the potential for long-term toxicities associated with these therapies and the field must develop new strategies to increase potency and efficacy while individualizing the selection of patients to those most likely to respond to treatment. Novel agents and modalities of therapy are also being explored. This review will discuss the current landscape and describe the avenues for growth in the field currently being explored.

## Introduction

Targeted radionuclide therapy plays an increasingly important role in management of neuroendocrine tumors. Currently available treatments include Lu-177 DOTATATE which is approved for advanced gastroenteropancreatic neuroendocrine tumors (GEP-NETs), and I-131 meta-iodobenzylguanidine (MIBG) which is approved for treatment of advanced pheochromocytomas/paragangliomas (1, 2). Lu-177 DOTATATE is a form of peptide receptor radionuclide therapy (PRRT) which targets somatostatin receptor (SSTR) subtypes overexpressed by most well-differentiated NETs. I-131 MIBG relies on the norepinephrine transporter mechanism which takes up amines in tissues derived from the neural crest such as the adrenal medulla and sympathetic nervous system (3).

Peptide receptor radionuclide therapy (PRRT) is a targeted systemic therapy that uses radiolabeled peptides to deliver cytotoxic radiation levels directly to tumors that overexpress specific receptors (4). This systemic administration of targeted radiopharmaceutical delivers therapeutic doses of radiation to specific disease sites while minimizing the radiation effect on healthy tissue.

In addition, several new therapies are currently being developed preclinically, and currently approved therapies are now being explored in other indications to see whether they can benefit patient populations with unmet needs. The changing treatment landscape of neuroendocrine tumors raises questions regarding optimal dosing, patient selection, role of combination therapy and sequencing of treatments.

## Neuroendocrine neoplasms

Neuroendocrine neoplasms (NEN) represent a heterogeneous family of cancers that can arise from various organ systems, primarily in the gastroenteropancreatic tract and lungs (5). The incidence of diagnoses has increased significantly in the last three decades (6). NEN biology, primarily defined by tumor grade and differentiation, dictates available therapies. Several treatment options are available for well-differentiated NETs, including somatostatin analogs, targeted therapies with mTOR inhibitors (everolimus), angiogenesis inhibitors (sunitinib), and oral chemotherapy agents (capecitabine/temozolomide) (7–9). In addition, clinical trials have explored several other therapies, including pazopanib, lenvatinib, surufatinib, and immunotherapy with checkpoint inhibitors, among other treatments (10–22). Poorly differentiated neuroendocrine carcinomas represent a different biology and are treated more aggressively with cytotoxic chemotherapy, primarily with platinum-based regimens (23–26). Therapy with radiolabeled somatostatin analogs, broadly encompassed under the class of PRRT, represents the newest treatment that has been approved for the GEP-NET patient population.

Data on therapeutic radionuclides in neuroendocrine neoplasms began with In-111-labeled octreotide, a weakly cytotoxic radiolabeled somatostatin analog (SSA). β-emitting isotopes were subsequently developed with the potential to elicit a more significant tumor response in patients with well-differentiated GEP-NETs that express SSTRs (27–29). Y-90ttrium (Y-90) and Lu-177-based radiolabeled SSAs (e.g., Y-90 DOTATOC and Lu-177 DOTATATE) have shown evidence of safety and efficacy in multiple studies with objective response rates ranging from approximately 15% to 40% and long median durations of progression-free survival (PFS), typically exceeding 2 years (30–38). The NETTER-1 trial was the first prospective phase III trial evaluating Lu-177 DOTATATE versus high-dose octreotide in patients with advanced small intestinal (midgut) NETs (34). The primary endpoint of the study was met with a 79% improvement in PFS. On final overall survival (OS) analysis, a secondary endpoint, median overall survival (OS) improved from 36.3 months on the high dose octreotide arm to 48 months on the Lu-177 DOTATATE arm (39). However, this result was not statistically significant, likely due to lack of power to detect OS differences and the effects of crossover after progression on high-dose octreotide. Toxicity with Lu-177 DOTATATE arm has generally been mild, with most side effects limited to minor nausea, fatigue, and reversible myelosuppression (38, 40–44). The risk of treatment-related myelodysplastic syndrome (MDS) or acute myeloid leukemia (AML) is estimated to be 2-3% and typically develops more than 2 years after completion of therapy (34, 39, 40). Sequential or combination therapy with alkylating-based chemotherapy agents may result in a higher risk of developing a long-term hematologic toxicity, with some series reporting up to a 10-20% risk (43–50). Renal toxicity has been negligible, likely due to the nephroprotective amino-acid infusion administered alongside the PRRT (42, 51–53). Recent data point to risk of severe, and often irreversible bowel obstruction among patients with extensive peritoneal or mesenteric disease receiving Lu-177 DOTATATE (54, 55). Data from the NETTER-1 study as well as a large Dutch cohort analysis of 610 NET patients led to approval of Lu-177 DOTATATE for patients with progressive, SSTR positive GEP-NETs.

Limited data is available in patients with lung NENs, partly due to typically heterogeneous uptake on SSTR imaging in this patient population (30, 31, 56–60). Data are available from small studies and phase II studies, showing mildly inferior PFS results compared to patients with GEP-NETs.

## Medullary thyroid cancer

Medullary thyroid cancer (MTC) is a neuroendocrine tumor of the parafollicular thyroid cells that can occur sporadically or as a component of multiple endocrine neoplasia (MEN) type 2 (61). MTCs secrete calcitonin and carcinoembryonic antigen (CEA). The primary systemic therapies approved for advanced MTC include tyrosine kinase inhibitors (TKIs), primarily targeting RET mutations, including selepercatinib, and pralsetinib, and multi-kinase inhibitors such as vandetanib and cabozantinib (62–78). Several others, sorafenib, sunitinib, and lenvatinib have also been studied (79–81). Due to the expression of CEA on MTC cells, radiolabeled anti-CEA monoclonal antibodies were developed. In an initial phase I trial of an anti-CEA hMN-14 x m734 bispecific antibody (BsMAb) and I-131 di-diethylenetriamine pentaacetic acid (DTPA)-indium hapten, 9 MTC patients were enrolled, and another 29 in a phase II trial later on (82). Ultimately, the drug has not been further developed in this patient population, likely due to the significant hematologic toxicities and lack of improvement in overall survival, despite its ability to induce long-term disease stabilization.

A retrospective study of 21 MTC patients who received treatment with Y-90 DOTATOC reported a radiographic disease control rate (DCR) of 67% (10% complete response (CR), 57% stable disease (SD)) (83). Biochemically (calcitonin and CEA levels), the disease control rate was 43% and the duration of response ranged from 3-40 months. Another retrospective analysis of 10 MTC patients treated with Lu-177 DOTATATE showed only 4 patients with disease stabilization at first follow-up (84). This analysis evaluated the percentage of patients at their institution with positive In-111 pentetreotide (OctreoScan) and found that most patients (89%) had low uptake on scans, and the remaining patients had only intermediate uptake (Krenning score grade 2). The patients in this study who had disease stabilization were characterized as those with uptake ≥ grade 3 on OctreoScan. Both studies have shown that PRRT in this patient population is unlikely to be effective in most patients and should be limited to those with high uptake on SSTR imaging (Cu-64 or Ga-68 PET DOTATATE) and without any other treatment options (85).

## Pheochromocytomas and paraganglioma

Pheochromocytomas and paragangliomas are catecholamine-secreting neuroendocrine tumors that arise from chromaffin cells of either the adrenal medulla (pheochromocytomas) or neuroendocrine cells of the extra-adrenal autonomic paraganglioma (paragangliomas) (86). Most are benign, however approximately 10% of pheochromocytomas and 25% of paragangliomas are malignant.

For patients with distant metastases, systemic therapies can include somatostatin analogs such as octreotide or lanreotide (although benefit is unproven), systemic chemotherapy with cyclophosphamide, vincristine and dacarbazine (CVD) or temozolomide, sunitinib, or radionuclide therapy with beta-emitting isotopes iobenguane I-131 MIBG (for patients with MIBG positive scans) or PRRT with Lu-177 DOTATATE (for patients with positive SSTR imaging) (87–94).

Iobenguane I-131 MIBG is a I-131 labeled radiopharmaceutical that is similar in structure to the neurotransmitter norepinephrine that is taken up by the norepinephrine transporter in adrenergic nerve terminals and accumulates in innervated adrenergic tissues as well as tumors of neural crest origin. Pheochromocytomas and paragangliomas express high norepinephrine transporter levels on cell surfaces. Patients must have positive metaiobenzeneguanidine (MIBG) scans to be eligible for treatment.

I-131 MIBG is the first FDA approved therapy for this patient population and is typically administered as a total of 2 doses, minimum of 90 days apart, at a weight-based dose of 8 mCi/kg for patients who weigh less than 62.5kg and a flat dose of 500 mCi for patients who weigh >62.5kg. Several small case series showed objective response rates of around 30% and stability in 40% of patients receiving treatment. A large phase II trial of 81 patients led to FDA approval of the drug (95). Patients were assigned to receive 2 dosimetric doses of MIBG, followed by up to 2 therapeutic doses, then followed for 12 months for efficacy and 4 years for overall survival. The study’s primary endpoint was at least 50% reduction in all antihypertensive medications lasting ≥ 6 months from the beginning of the efficacy period. Secondary endpoints included objective response rate (ORR) and OS. 68 patients received at least one therapeutic dose and 50 received both doses. Primary endpoint was met by 25% of those who received one therapeutic dose and 32% of those who received both doses, meeting pre-specified protocol criteria for a positive study. In addition, 23% of patients who received one dose and 30% of those who received two doses achieved a partial response (PR) per RECIST 1.1. Median OS was 36.7 months, and five-year OS was 64%. Most common treatment-related toxicities included significant myelosuppression, with ≥ grade 3 neutropenia and thrombocytopenia in 87% of patients. 4 patients required autologous hematopoietic cell rescue and 2 patients developed MDS. 2 patients each developed grade 4 acute respiratory distress and cryptogenic organizing pneumonia.

Several retrospective studies of I-131 MIBG have also been reported (96). For example, one study of 125 patients with metastatic pheochromocytoma/paraganglioma reported a median survival post-treatment of 4 years, an ORR of 34%, and DCR of 86%; the median PFS was two years. In addition, 59% of patients achieved a biochemical response, with the median time to laboratory progression of 2.8 years.

PRRT with Lu-177 DOTATATE or Y-90 DOTATOC has been evaluated in retrospective studies and small case reports/case series in patients with SSTR-expressing pheochromocytomas/paragangliomas (92, 97). One study evaluated the outcomes of 28 patients who received Y-90 DOTATOC alone or with Lu-177 DOTATATE (88). Two patients experienced a PR; overall DCR was 71%, of whom 50% maintained their response at 19 months mean follow-up. Another study reported the outcomes of 30 patients who received 4 cycles of Lu-177 DOTATATE. 23% of patients achieved a PR per RECIST 1.1, and stable disease in 67%. Median PFS for patients with parasympathetic paragangliomas was 91 months, 13 months in patients with sympathetic paragangliomas, and 10 months in patients with pheochromocytoma. 20% of patients experienced ≥ grade 3 hematologic toxicities, and 2 patients had reversible cardiac failure following catecholamine release. Patients must be monitored and pre-treated with alpha blockade therapy if functional tumors are present to prevent hypertensive crises after treatment with any of these types of treatment (98).

## Future of PRRT and novel approaches to treatment

### Dosimetry and predictive biomarkers

Individualized dosimetry strategies have been proposed and primarily focus on minimizing renal and bone marrow exposure while maximizing the potential for anti-tumor activity (42, 51, 99–103). Unfortunately, these approaches have been limited by the lack of standardization in dosimetric methodology, lack of clear thresholds for renal exposure, and difficulties in accurately measuring bone marrow exposure.

The European Association of Nuclear Medicine (EANM) recommends dosimetry, where feasible, in patients receiving radiolabeled somatostatin-receptor ligands and radiolabeled MIBG therapy, particularly for patients with larger tumor volume, due to the relatively limited spatial resolution of MIBG imaging (104).

Biomarkers to predict responders to PRRT are being investigated. Ki67% has been noted to significantly impact OS in both GEP NETs and pheochromocytoma/paraganglioma but is likely more of a prognostic than predictive factor (105–107). Tumor imaging with baseline MIBG scans, PET DOTATATE, and FDG-PET scans can help delineate who is likely to respond (or be eligible). Obtaining baseline FDG-PET scans, particularly in patients with the higher-grade disease, can help delineate the avidity of disease, and for patients with higher avidity on FDG-PET, PRRT is likely to be less effective (108). It is also essential to confirm that all lesions express SSTRs, particularly in patients with higher-grade disease. A genomic signature (blood and tumor-based NET transcript assay – PRRT Predictive Quotient or PPQ) to identify NET responders to PRRT with Lu-177 DOTATATE has been developed and is currently being evaluated in a large, multi-center, clinical trial (109–111).

A blood-based RNA assay was developed to identify gene expression differences in patients receiving I-131 MIBG and demonstrated the ability to use biodosimetric gene expression panels as predictive biomarkers of internal exposure and differentiate exposed individuals up to 15 days after exposure to treatment (112). However, data on predictive biomarkers for all radioisotopes are limited, and further research is necessary to advance this field.

### PRRT retreatment

Retreatment with Lu-177 DOTATATE beyond the standard 4 cycles is often recommended for patients who benefit from initial treatment (52, 113, 114). The lifetime maximum of standard dose Lu-177 DOTATATE (200mCi per treatment) is roughly 6-8 cycles. Retreatment is typically reserved for patients who initially experience at least 12 months of disease response or stability following completion of initial treatment. In one cohort of 168 patients retreated with 2 additional cycles of Lu-177 DOTATATE, the median PFS was 14.6 months (115). Similarly, 131I-MIBG is typically given as a set of 2 cycles, 3 months apart, but retreatment with additional cycles, usually at 6-month intervals after treatment with the initial regimen has been reported; however, the optimal dosimetry is not yet established (116). Reports have described cumulative doses of 557 – 2322 mCi with individual doses between 100 – 200 mCi.

### Intra-arterial and liver directed therapy

For patients with liver dominant disease, intra-hepatic administration of PRRT provides a unique opportunity to target progressing disease with higher concentrations of the therapy and reduce systemic toxicity. This approach has been evaluated in patients with GEP-NETs retrospectively, showing that patients with liver-limited disease had longer median PFS and OS than those across the entire cohort of patients (33.4 and 75.8 months vs. 29 and 70 months, respectively) (37, 117).

### Combination therapy

Several studies have explored Lu-177 DOTATATE and Y-90 DOTATOC combined with different cytotoxic agents: primarily capecitabine and temozolomide (49, 50, 118, 119). Patients were either treated concurrently, sequentially, or in one instance, in a “sandwich” fashion, where patients received 2 cycles of PRRT followed by cycles of chemotherapy before they received the remaining PRRT doses. Response rates tended to be higher in these patient populations, with ORR as high as 57%, however, long-term follow-up of these cohorts revealed higher rates of treatment-related MDS or acute leukemia and no significant improvement in PFS or OS compared to PRRT alone. A recent study of 49 patients who received PRRT and chemotherapy with capecitabine/temozolomide reported a 10% risk of developing t-AML/AL, a significantly higher risk than PRRT alone (120).

Other avenues being explored in primarily the GEP-NET population are combinations with mTOR inhibitors, radiation sensitizers (such as cell signaling inhibitors, DNA damage repair inhibitors, and DNA damage inducers), and tandem PRRT with multiple radioisotopes (121–127). The majority of research thus far has been preclinical in mouse models and xenografts, however a few clinical trials have recently begun enrollment and are exploring the combinations of radiosensitizers and DNA-damage-repair inhibitors in combination with PRRT. **Table 1** summarizes ongoing and currently recruiting clinical trials.

**Table 1:** Ongoing clinical trials with PRRT.

Study Title	Study NCT Number	Phase	Treatment	Patient population
Lu-177 DOTATATE (Lutathera) in Combination with Olaparib in Inoperable Gastroenteropancreatic Neuroendocrine Tumors (GEP-NET)	NCT04086485	I/II	Lu-177 DOTATATE + Olaparib	Inoperable or metastatic GEP-NETs
Pembrolizumab With Liver-Directed or Peptide Receptor Radionuclide Therapy for Neuroendocrine Tumors and Liver Metastases	NCT03457948	II	Lu-177 DOTATATE or liver-directed therapies + Pembrolizumab	G1-G3 NETs
Personalized CAPTEM Radiopeptide Therapy of Advanced, Non-resectable Neuroendocrine Cancer	NCT04194125	II	Y-90 DOTATOC + CAPTEM	G1-G2 GEP-NETs
Peptide Receptor Radionuclide Therapy (PRRT) in Tumors with High Expression of Somatostatin Receptors (Phase 2) (FENET-2016)	NCT04790708	II	Lu-177 DOTATOC ± Y-90 DOTATOC (monotherapy arms and combination arms; retreatment arms of individual therapy)	GEP-NETs, Bronchial NETs, Pheochromocytoma, Paraganglioma, Neuroblastoma, other NETs (skin, thyroid, parathyroid origin), NETs of unknown primary
Targeted Alpha-emitter Therapy of PRRT Naive Neuroendocrine Tumor Patients (ALPHAMEDIX02)	NCT05153772	II	Pb-212 DOTAMTATE	NENs
Intra-arterial Hepatic (IAH) Infusion of Radiolabeled Somatostatin Analogs in GEP-NET Patients With Dominant Liver Metastases (LUTARTERIAL)	NCT04837885	II	Lu-177 DOTATATE via intrahepatic injection	GEP-NETs
Combined Beta- Plus Auger Electron Therapy Using a Novel Somatostatin Receptor Subtype 2 Antagonist Labelled with Terbium-161 (Tb-161 DOTA-LM3) (Beta plus)	NCT05359146	I	Lu-177 DOTATOC and Tb-161 DOTA-LM3	GEP-NENs (G1 and G2 only)

### Alpha-emitting isotopes and somatostatin-receptor antagonists

Alpha-emitters allow for more precise targeted therapy due to their shorter penetration range and higher linear energy (102, 128). A phase I study of Pb-212 DOTAMTATE reported a response rate of 80% among PRRT naïve patients on the highest dose of the drug (129). In addition, a cohort study with Ac-225 DOTATATE reported a >44% ORR in GEP-NET patients who had previously received Lu-177 DOTATATE (130). These data are promising, and further development is ongoing, with several clinical trials currently recruiting patients.

SSTR antagonists can occupy more binding sites and have lower dissociation rates than somatostatin analogs, which can lead to higher tumor uptake and a lower risk of radiation to healthy surrounding tissue (131). Several studies are ongoing in this arena in patients with NETs; however, no final data has been published. Lu-177 OPS201 (also known as Lu-177 DOTA-JR11 or Satoreotide tetraxetan) is one of the antagonists that is now being tested in clinical trials NCT02592707, NCT03773133 (132).

## Conclusions

Radionuclide therapies represent a significant advancement in the therapeutic landscape for patients with endocrine-related cancers with limited availability of FDA-approved systemic therapies for advanced, progressive disease. The field has seen significantly more advances in patients with GEP-NETs and pheochromocytoma/paraganglioma, with the approval of I-131 MIBG and Lu-177 DOTATATE. Despite these advances, there remains a great deal of research to be done on other cancer types and indications. With increased knowledge of potential long-term toxicities associated with these therapies, we must develop strategies to increase the potency and efficacy while also individualizing the selection of patients who will most likely respond to treatment by employing biomarkers and imaging studies. Novel agents with α-emitters and SSTR antagonists, combination treatments (whether with other radionuclides or systemic targeted/chemotherapies), radiosensitizers, or unique modalities of treatment administration such as intra-arterial therapies are all promising advances in the field, however, most of the effort is currently focused on GEP-NETs. Clinical trials focusing on refining this therapy in other endocrine cancers will be important to expand impact of radiopharmaceuticals.

## Author contributions

TA-T conducted the literature search and wrote the first draft of the manuscript. TA-T, GE-H contributed to conception and design of the manuscript. JS, J-JH, and GE-H provided expert feedback and edits to content. All authors contributed to the article and approved the submitted version.
